# Acute liver injury secondary to hemophagocytic lymphohistiocytosis triggered by Epstein–Barr virus infection

**DOI:** 10.1002/jgh3.12439

**Published:** 2020-10-22

**Authors:** Aiji Hattori, Yasuhiko Hamada, Hiroyuki Kawabata, Kyosuke Tanaka

**Affiliations:** ^1^ Department of Gastroenterology Saiseikai Matsusaka General Hospital Matsusaka Japan; ^2^ Department of Gastroenterology and Hepatology Mie University Hospital Tsu Japan; ^3^ Department of Endoscopy Mie University Hospital Tsu Japan

**Keywords:** hepatic inflammation, viral hepatitis, virology

## Abstract

We present a 23‐year‐old man with hemophagocytic lymphohistiocytosis (HLH) triggered by Epstein–Bar virus (EBV) infection. This patient presented with persistent fever and acute liver injury 6 weeks after having an infectious mononucleosis associated with EBV infection. He had hypofibrinogenemia, hyperferritinemia, increased soluble interleukin‐2 receptor, elevated prothrombin time, and pancytopenia. Bone marrow examination for evaluation of pancytopenia revealed that macrophages had phagocytosed mature erythrocytes. Based on these findings, we suspected an HLH triggered by EBV infection (EBV‐HLH). To distinguish from HLH triggered by malignant lymphomas accompanying EBV infection, we performed a percutaneous liver biopsy, which revealed that atypical T‐lymphocytes had infiltrated the liver tissues. The T‐lymphocytes were positive for EBV‐encoded RNA in situ hybridization, and no distinct monoclonal T‐cell receptor chain gene rearrangement was detected. These findings indicated EBV hepatitis and, accordingly, malignant lymphoma was ruled out. We finally made a diagnosis of EBV‐HLH. The patient was treated with corticosteroid and etoposide, according to HLH‐2004 guideline recommendations, and the patient's symptoms and laboratory values improved. After that, he experienced no recurrence. Prompt recognition and initiation of treatment remains the key to the survival of patients with EBV‐HLH, and the liver biopsy was helpful in making the diagnosis.

## Graphical abstract

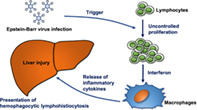

This article presents a case of hemophagocytic lymphohistiocytosis (HLH) triggered by Epstein–Barr virus (EBV) infection. HLH triggered by EBV infection is a rare but life‐threatening syndrome, causing liver injury. Clinicians may encounter patients with HLH and should have a general understanding of the condition.

## Introduction

Hemophagocytic lymphohistiocytosis (HLH) is a very rare but life‐threatening syndrome of hyperactive immune activation and is characterized by high levels of cytokines, reflecting activation of T‐cells and macrophages.^1^ The diagnosis of HLH is currently based on the diagnostic criteria of HLH‐2004,^2^ and at least five of the eight diagnostic criteria should be fulfilled for the diagnosis: (i) high fever, (ii) splenomegaly, (iii) cytopenias involving at least two of three lineages in the peripheral blood, (iv) hypertriglyceridemia or hypofibrinogenemia, (v) hemophagocytosis, (vi) low or absent natural killer (NK) cell activity, (vii) hyperferritinemia, and (viii) an increased soluble interleukin‐2 receptor (sIL‐2R) level.

The diagnosis of HLH is usually challenging because it does not present any specific clinical findings.^3^, ^4^ Here, we describe an adult case of acute liver injury secondary to HLH triggered by Epstein–Barr virus (EBV) infection, and a liver biopsy was useful to make the definite diagnosis.

Informed consent was obtained from the patient for the publication of his information and imaging.

## Case Report

**Figure 1:**
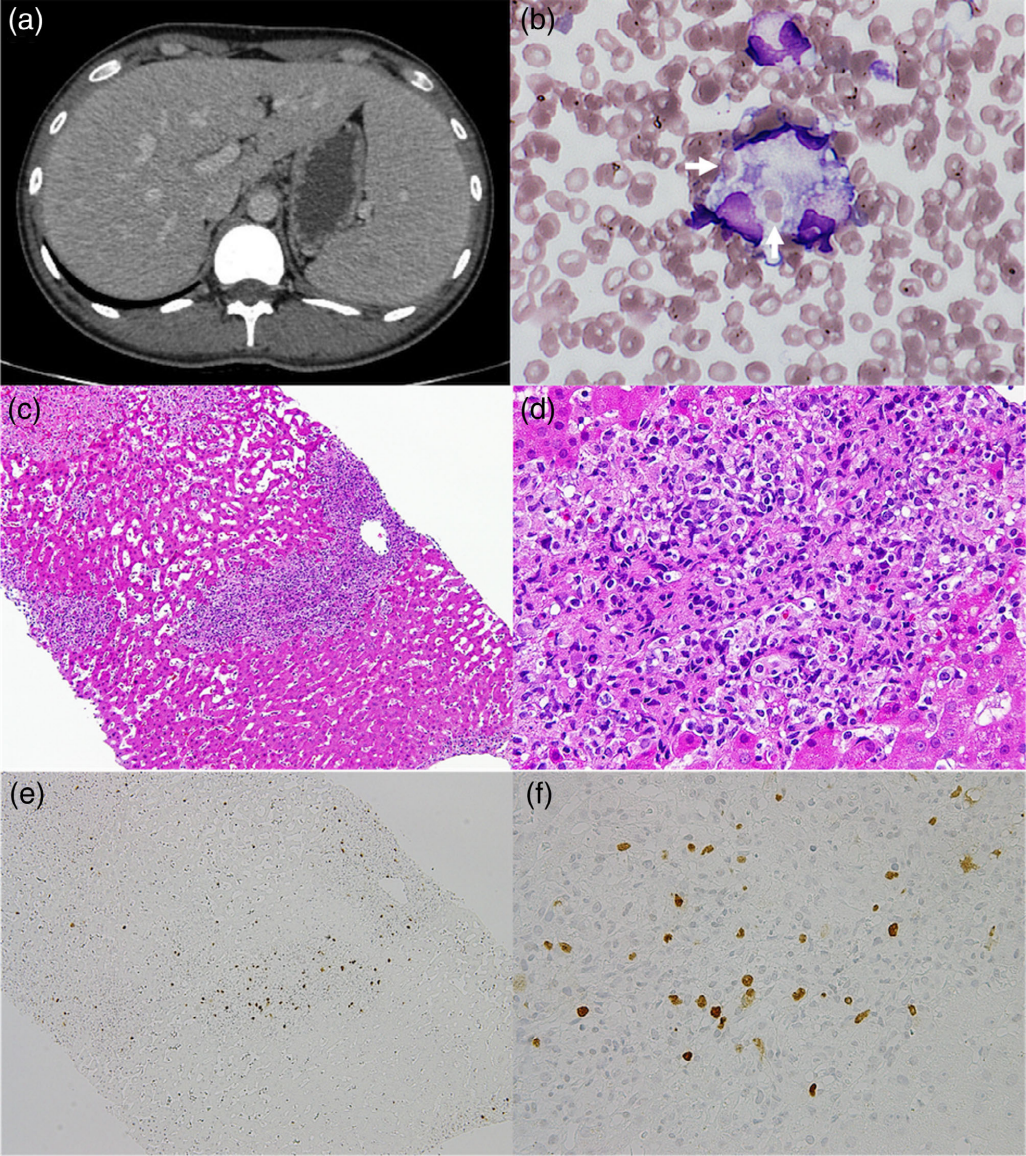
Computed tomography showed hepatosplenomegaly (a). Bone marrow examination showed that macrophages had phagocytosed mature erythrocytes (b, 400×, arrows). Liver biopsy showed that non‐neoplastic T‐lymphocytes markedly infiltrated Glisson's capsules and lobules (c, 100×; d, 200×). Epstein–Barr virus (EBV)‐encoded small RNA in situ hybridization stain showed that the T‐lymphocytes were infected with EBV (e, 100×; f, 200×).

A 23‐year‐old non‐alcoholic man without past medical history presented with a high fever of 38.0°C and sore throat. He was diagnosed with infectious mononucleosis by EBV infection on serological testing, showing positivity for the EBV‐IgM viral capsid antigen but negativity for the EBV‐IgG viral capsid antigen and early antigen. His symptoms had improved with supportive care. However, 6 weeks later, he presented with a persistent fever of 40.0°C. His consciousness level was clear. Computed tomography scans showed a hepatosplenomegaly but no fatty liver or lymphadenopathy (Fig. 1a). Laboratory results showed elevated liver function tests (aspartate transaminase 696 IU/L, alanine transaminase 336 IU/L, lactate dehydrogenase 1862 IU/L, alkaline phosphatase 630 IU/L, and total bilirubin 4.9 mg/day), hypofibrinogenemia (100 mg/dL), increased ferritin (5833 ng/mL), increased sIL‐2R (3785 U/mL), elevated prothrombin time (56%), and pancytopenia (white blood cell count 1200/μL, hemoglobin 8.9 g/dL, platelet 21 × 10^3^/μL). All autoimmune testing was negative, and serological testing for hepatitis B and C viruses and human immunodeficiency virus (HIV) was also negative. Bone marrow examination for evaluation of pancytopenia revealed that macrophages had phagocytosed mature erythrocytes (Fig. 1b, arrows). Based on these findings, we suspected an HLH triggered by EBV infection (EBV‐HLH). To distinguish from HLH triggered by malignant lymphomas accompanying EBV infection, we performed a percutaneous liver biopsy. The biopsy revealed that atypical T‐lymphocytes (CD3+, CD8+, CD56−, and CD20−) had markedly infiltrated the hepatic lobules, Glisson's capsules, and sinusoids (Fig. 1c, 100×, d, 200×). The T‐lymphocytes were positive for EBV‐encoded RNA in situ hybridization (Fig. 1e,f). No distinct monoclonal T‐cell receptor chain gene rearrangement was detected. Collectively, these findings indicated an EBV hepatitis, and malignant lymphoma was ruled out. The number of copies of EBV DNA in the peripheral blood had increased (1.3 × 10^6^ copies/mL), and he met seven of the eight diagnostic criteria of the HLH‐2004. Therefore, we finally made a diagnosis of EBV‐HLH. The patient was treated with corticosteroid and etoposide according to the HLH‐2004 guideline recommendations. The treatments went well, and the patient was discharged. Thereafter, he remained asymptomatic, and there was no recurrence.

## Discussion

Adult HLH is divided into familial and acquired forms, with 90% of adult cases being acquired HLH.^1^ Various diseases, including malignancies, autoimmune diseases, and infections, may cause HLH. Thus, the heterogeneity of its initial symptoms, such as persistent fever, hepatosplenomegaly, and liver injury, may make it difficult to reach a prompt diagnosis.^5^ When evaluating an adult for persistent fever and severe systemic illness, HLH should be in the differential diagnosis, particularly after a viral illness.

The basis for the treatment of HLH requires a threefold approach.^1^ First, supportive therapies are essential because of the life‐threatening presentation. Second, the elimination of triggers (e.g. infection and malignancy) is crucial to remove the stimulus that initiates abnormal activation of the immune system. Third, suppression of the inflammatory response and neoplastic cell proliferation by immunosuppressive and cytotoxic drugs (glucocorticoid, cyclosporine, and etoposide) is necessary.^6^

EBV infection is the most common viral trigger, and it accounts for approximately 15% of acquired HLH in adults,^1^ and EBV‐HLH can sometimes present with acute liver injury as the predominant feature. Here, for the definitive diagnosis of EBV‐HLH, the clinical symptoms, imaging, and laboratory results fulfilled not only the HLH‐2004 criteria but also featured high values of EBV DNA copies in peripheral blood and/or a number of cells containing EBV‐encoded small RNA in the tissues.^4^ On the other hand, more than a quarter of HLH patients with EBV infection are categorized as HLH triggered by malignant lymphomas associated with EBV infection.^4^ Thus, for the diagnosis of EBV‐HLH, biopsy and pathological confirmation are needed to exclude the lymphoma cases. In our case, liver biopsy was undertaken because of the acute liver injury, and the biopsy revealed that the possibility of lymphomas was unlikely because monoclonal T‐cell receptor chain gene rearrangement was not detected. Thus, we could reach a prompt diagnosis of EBV‐HLH and provide treatment.

EBV‐HLH is associated with a poor prognosis in secondary HLH, excluding malignancy‐associated HLH.^7^ Patients with EBV‐HLH have similar responses to treatment during the early period, but many relapse thereafter, finally resulting in these patients having significantly inferior overall survival rates and higher cumulative incidence rates of progression, compared to patients with HLH triggered by other causes. Thus, it is important to decide on early salvage treatment, such as allogeneic hematopoietic cell transplantation, for poor responders with EBV‐HLH.^7^

In conclusion, early recognition and initiation of HLH‐directed therapy remains a key factor for a successful outcome in this life‐threatening condition, and a liver biopsy may be helpful in reaching a prompt diagnosis of EBV‐HLH.
